# The dosing conundrum of emicizumab: To waste product or not?

**DOI:** 10.1016/j.rpth.2023.100087

**Published:** 2023-02-16

**Authors:** Guy Young

**Affiliations:** Cancer and Blood Disorders Institute, Children’s Hospital Los Angeles, University of Southern California Keck School of Medicine, Los Angeles, California, USA

Historically (and still today) the common dispensing practice for factor therapy (in countries without significant limitations to factor availability) is to round the prescribed dose up or down 10%, which is done due to the combination of weight-based dosing and limited vial size availability. This practice has served the community well, making the filling of prescriptions simpler and in some cases reducing wastage of a very expensive product. The study by Donners et al. [1] builds on this philosophy, extending it to emicizumab though without limiting the rounding of the dose to ±10%, and suggests that using the entire vial results in a 9% reduced wastage of product, which can translate into a substantial cost savings. Furthermore, in the study cohort, although very small, there were no safety issues. Can this approach be broadly applied and what are the strengths and limitations of this approach?

Before answering this question, a basic understanding of emicizumab dosing and dispensing is required. First, emicizumab dosing begins with 4 weekly bolus doses of 3 mg/kg, which are then followed by maintenance doses consisting of 3 strict weight-based options for patients of all ages and all weights (1.5 mg/kg per week, 3 mg/kg every 2 weeks, or 6 mg/kg every 4 weeks [the total dose over 4 weeks is the same regardless or regimen]). Second, emicizumab is only available in 4 vial sizes; however, the smallest vial size (30 mg) has a different concentration (30 mg/mL vs 150 mg/mL for the other 3) and cannot be combined in 1 syringe with the other vial sizes [2]. Immediately it becomes apparent, as the authors duly noted, that only on rare occasions, eg, a 55-kg patient on a regimen of 3 mg/kg every 2 weeks (dose of 165 mg equating to a 105-mg vial and a 60-mg vial), does the patient’s dose match 1 or a combination of exact vial sizes. This is what creates the conundrum of prescribing (and administering) the exact dose vs using the whole vial. When one reviews the detailed pharmacokinetic data from the HAVEN 1-4 trials [[3], [4], [5], [6]], clearly there are ranges of levels around the mean level; however, they are not exceptionally broad. Although no one would argue (using the example above) that a 54- or 56-kg patient can receive the 165-mg dose, the issue becomes more concerning for a 25-kg patient on a biweekly (3 mg/kg) regimen where the dose should be 75 mg. Using the full 60-mg vial would likely result in lower levels and could lead to reduced efficacy, whereas using the full 105-mg vial results in a rounding up of the dose of 40%. Although one could argue that this should not be a problem, and the phase 1 study of emicizumab [7] used doses of 3 mg/kg per week albeit in only a few patients, what if an adverse event such as a thrombosis does happen with this off-label dose? In the litigious United States, this could result (however unfair it may seem) in a lawsuit against the prescribing physician, and although this may on the surface seem defensible, as one who has served as an expert witness in lawsuits, I can assure you this would be problematic. The plaintiff’s lawyer would quote the prescribing information, the clinical trials, and point to the absence of evidence for using a 40% “overdose” as well as the role of the Food & Drug Administration and could make a very convincing argument to a judge or jury that the “overdose” harmed the patient. On the other hand, the authors rightly point out the simplicity of full vial dosing and make an economic argument as well for this approach, even if the savings of 9% seems somewhat modest.

So, what can be done about this in general, and what should prescribers consider given the data from this study? For the first question, it seems to me that only the manufacturer can address this. Options could among other things include offering several more vial sizes between the ones currently offered. Although this seems to be a simple fix, apparently it is quite a complicated manufacturing issue, and as I am no expert in this area, only Roche can address the community about this. Another option that would rely on the manufacturer is to have a precise dosing device that could dispense the exact amount of drug, but at this point in the life cycle of emicizumab, that seems even less likely than the prospect of more vial sizes. The other option is for academia (perhaps in partnership/sponsorship from the manufacturer) to conduct a phase 4 study evaluating different dosing approaches such as dose bands, ie, all patients between 10 and 20 kg, 20 and 30 kg, etc. receive the same dose. Certainly, this would significantly simplify dosing and eliminate most if not all the drug wastage. Regarding the second question of what prescribers can/should do now, again, there are different options. The first (and safest both medically and medico-legally) is to continue to prescribe and administer the drug according to the dose per the prescribing information with minimal rounding such as in situations like the one described above where rounding is a few percent off the exact dose. In our institution, we have made a table of suggested dosing regimens by weight to minimize wastage; however, it may necessitate patients using a dosing frequency or injection volume they may not prefer (Table). We do not strictly follow this approach, and it is only a suggested recommendation with the main goal of minimizing wastage. The second (as some are already doing) is to round to the nearest whole vial regardless of the percent increase/decrease from the exact dose with no laboratory monitoring. The third approach would be to follow the author’s approach, which would involve the same but with the added safety margin of checking emicizumab levels. Unfortunately, for nearly all treating physicians, laboratory assays that measure the drug concentration are not available. Assays that closely (the *r*^*2*^ diagnostics emicizumab calibrator [8]) or “less closely” (human chromogenic factor VIII level [9]) approximate an emicizumab level, although more readily accessed, are still not available to most prescribers. Thus, following the author’s approach is generally not feasible.

**Table:** The ideal maintenance dosing regimen by weight to minimize discarding (wasting product) product (*italics* represent dosing regimens that utilize the full vial).

Weight (kg)	Dose regimen	Dose	Vial to be used	Volume (mL)
*5*	*6 mg/kg every 4 wk*	*30 mg*	*30 mg*	*1*
*10*	*6 mg/kg every 4 wk*	*60 mg*	*60 mg*	*0.4*
15	3 mg/kg every 2 wk 6 mg/kg every 4 wk	45 mg 90 mg	60 mg 105 mg	0.3 0.6
*20*	*3 mg/kg every 2 wk* *6 mg/kg every 4 wk*	*60 mg* *120 mg*	*60 mg* *60 mg*	*0.4* *0.8*
*25*	*6 mg/kg every 4 wk*	*150 mg*	*150 mg*	*1*
30	6 mg/kg every 4 wk	180 mg	150 mg + 30 mg	1 + 1
*35*	*3 mg/kg every 2 wk* *6 mg/kg every 4 wk*	*105 mg* *210 mg*	*105 mg* *105 mg + 105 mg*	*0.7* *0.7 + 0.7*
*40*	*1.5 mg/kg/wk* *3 mg/kg every 2 wk*	*60 mg* *120 mg*	*60 mg* *60 mg + 60 mg*	*0.4* *0.8*
45	1.5 mg/kg/wk 3 mg/kg every 2 wk 6 mg/kg every 4 wk	67.5 mg 130 mg 270 mg	105 mg 150 mg 150 mg + 150 mg	0.45 0.9 1 + 0.8
*50*	*3 mg/kg every 2 wk* *6 mg/kg every 4 wk*	*150 mg* *300 mg*	*150 mg* *150 mg + 150 mg*	*1* *1 + 1*
*55*	*3 mg/kg every 2 wk*	*165 mg*	*105mg + 60 mg*	*0.7 + 0.4*
60	1.5 mg/kg/wk 3 mg/kg every 2 wk 6 mg/kg every wk	90 mg 180 mg 360 mg	105 mg 105 mg + 105 mg 150 mg × 2 + 60 mg	0.6 0.7 + 0.5 1 + 1 + 0.4
65	1.5 mg/kg/wk 3 mg/kg every 2 wk	97.5 mg 195 mg	105 mg 105 mg + 105 mg	0.65 0.7 + 0.65
*70*	*1.5 mg/kg/wk* *3 mg/kg every 2 wk*	*105 mg* *210 mg*	*105 mg* *105 mg + 105 mg*	*0.7* *0.7 + 0.7*
75	1.5 mg/kg/wk *6 mg/kg every 4 wk*	112.5 mg *450 mg*	150 mg *150 mg × 3*	0.75 *1 + 1 + 1*
80	1.5 mg/kg/wk 3 mg/kg every 2 wk	120 mg 240 mg	150 mg 150 mg + 105 mg	0.8 1 + 0.6
85	3 mg/kg every 2 wk 6 mg/kg every 4 wk	255 mg 510 mg	150 mg +105 mg 150 mg × 3 + 60 mg	1 + 0.7 1 + 1 + 1 + 0.4
90	1.5 mg/kg/wk 3 mg/kg every 2 wk	135 mg 270 mg	150 mg 150 mg + 150 mg	0.9 1 + 0.8
95	1.5 mg/kg/wk 3 mg/kg every 2 wk	142.5 mg 285 mg	150 mg 150 mg + 150 mg	0.95 1 + 0.9
*100*^a^	*1.5 mg/kg/wk* *3 mg/kg every 2 wk* *6 mg/kg every 4 wk*	*150 mg* *300 mg* *600 mg*	*150 mg* *150 mg + 150 mg* *150 mg × 4*	*1* *1 + 1* *1 + 1 + 1 + 1*

a For patients more than 100 kg, doses can be extrapolated further from the table.

In conclusion, there is a clear unmet need regarding emicizumab dosing regimens and available vial sizes resulting in wastage of the product as well as complicating the administration process, and simple and safe solutions are either not available or not optimal. Optimistically, newer agents currently in clinical trials are taking a different approach to dosing, including fixed dosing for fitusiran with 1 dose for those over 12 years and another for those less than 12 years [10], dosing bands by weight groups as with MIM8 [11], and precise weight-based dosing with a specialized injection device as with concizumab [12]. Until those agents become available and assuming they are safe and efficacious as expected, prescribers of emicizumab should carefully consider the options outlined above, including the rounding to the nearest whole vial favored by the authors who I commend for providing excellent data to support this approach, although with the caveats mentioned above.
